# Randomized Trial of Irrigation and Curetting for Cerumen Removal in Young Children

**DOI:** 10.3389/fped.2019.00216

**Published:** 2019-06-06

**Authors:** Timothy R. Shope, Cathy P. Chen, Hui Liu, Nader Shaikh

**Affiliations:** ^1^Division of General Academic Pediatrics, Department of Pediatrics, University of Pittsburgh School of Medicine, Children's Hospital of Pittsburgh of UPMC, Pittsburgh, PA, United States; ^2^University of Pittsburgh School of Medicine, Pittsburgh, PA, United States

**Keywords:** cerumen removal, ear wax removal, children, curette, irrigation

## Abstract

**Objectives:** To gather preliminary data on the effectiveness and feasibility of cerumen removal using three irrigation methods and a metal curette in young children.

**Study design:** Pilot study conducted as a randomized clinical trial of well and ill children age 6 months to 6 years with ≥25% cerumen occlusion in at least one ear. Children were stratified by age and randomized to one of four methods of cerumen removal: syringe with attached angiocath tubing, Elephant Ear Washer Bottle System^®^, OtoClear^®^ Spray Wash Kit, or metal curette. Clinicians, blinded from treatment assignment, assessed the degree of cerumen occlusion before and after the procedure. Outcomes included reduction in cerumen occlusion, successful removal, time until completion and parental satisfaction. Rules for stopping procedures were established *a priori*.

**Results:** Thirty-eight children underwent procedures (59 ears). There were no significant differences in reduction in cerumen and successful removal among the methods. Overall, 36 (61%) of 59 of procedures were successful. The syringe with angiocath tubing took the most time (*P* = 0.04) and resulted in the most stopped procedures (*P* < 0.01). Parental satisfaction scores were not significantly different.

**Conclusions:** Irrigation methods performed comparably to cerumen removal with curette; the SA method had drawbacks. Irrigation can be performed by non-clinicians, which is potentially a significant advantage. (Clinical trial registration: http://www.isrctn.com/ISRCTN74402562).

## Introduction

Cerumen impaction, present in about 1 in 10 children (1), can cause ear discomfort, reduced hearing and prevent the clinician from adequately visualizing the tympanic membrane (TM) to diagnose acute otitis media (AOM) and other conditions such as effusions, retraction pockets, foreign bodies, etc. Visualizing most of the TM is important to detect bulging, the most specific finding for AOM (2). Although incidence of AOM is highest in children age 6–24 months, very few children in this age group are included in studies of cerumen removal. Furthermore, no studies in any age group have compared the effectiveness of different methods of cerumen removal (1).

Methods for cerumen removal in the office setting include curetting, suction, irrigation, and cerumenolytics (1). Each method has advantages and disadvantages in terms of patient comfort, parental tolerance, required training, time, manpower needed, and expense. Cerumen removal by curette requires trained clinicians because of the technical skill required (3), whereas irrigation can be performed by nurses or medical assistants. Thus, if irrigation and curettage are equally effective irrigation may be preferred. The aim of this pilot study was to gather preliminary data on effectiveness, feasibility, and safety of curetting and three irrigation methods in young children with cerumen obstructing visualization of the TM.

## Methods

The Institutional Review Board of the University of Pittsburgh approved the study. We recruited children from Children's Hospital of Pittsburgh Primary Care Center, a residency teaching site with approximately 20 faculty pediatricians, between June and August 2016. Clinicians notified the research assistant when they saw a well or sick child, age 6 months to 6 years, with cerumen occluding ≥25% of at least one TM by otoscopy. We excluded children with otorrhea, hearing aids, or history of TM perforation, tympanostomy tubes, or otitis externa in the previous 2 weeks.

After obtaining written informed consent, children were randomized to one of four treatment groups: (1) irrigation using a 60-milliliter (mL) syringe with attached angiocath tubing; (2) irrigation using the Elephant Ear Washer Bottle System^®^ (permission granted, Doctor-Easy, Orange Park, Florida); (3) irrigation using the OtoClear^®^ Spray Wash Kit (permission granted, Bionix Medical Technologies, Toledo, Ohio); or 4) metal curette (Buck Ear Curette, 1.5 mm, Bausch and Lomb, Bridgewater, New Jersey). If a child had ≥25% cerumen occlusion in both ears the same procedure was used for each ear. Ears with ≤24% occlusion were not cleaned. It was not possible to blind subjects or parents to treatment assignment, but we blinded clinicians who assessed cerumen occlusion before and after cleaning. We randomized children using computer-generated blocks of four to generate equal treatment allocation within the following age strata: 6–23, 24–47, and 48–71 months. The randomization scheme was developed by a statistician prior to study initiation and the research assistant was unaware of block size. Treatment assignment was only revealed to investigators and patients after consent was signed.

### Cerumen Removal

Curetting (CU) was performed by an experienced clinician using a metal curette using the technique described by Shaikh et al. (4). Irrigation was performed by a medical assistant or nurse using a syringe with angiocath tubing (SA), Elephant Ear Washer Bottle System^®^ (EE), or OtoClear^®^ Spray Wash Kit (OC). We did not pretreat with a cerumenolytic because there is no apparent benefit over water (5). For the SA procedure, we used a 60-mL syringe coupled by Luer lock to a 23-gauge angiocath tubing cut off at 1.5 centimeters (cm). The needle and excess tubing were discarded. This length was chosen for safety because the average length of the external auditory canal in a newborn is 1.68 cm (6). The syringe was filled with warm water and a single stream of water was expressed through the angiocath tubing into the ear canal. The EE and OC systems are FDA-approved squirt-bottle irrigation systems with hand-held squeeze triggers that were attached to reservoirs filled 420 mL of warm water. The EE is equipped with 2 cm catheter-like tips that we cut to 1.5 cm to deliver a single stream of water into the ear canal. The OC delivers water via a plastic tip shaped like an ear speculum with three angled holes to direct streams to the walls of the ear canal. All irrigation methods allowed water, cerumen, and debris to continuously exit the ear canal. The medical assistant or nurse performing irrigation inspected the ear canal using an otoscope after approximately 100 mL, after removal of a large piece of wax, or if the child needed a break. After irrigation, the ear canal was dried by tipping the child's head allowing drainage out of each ear. If moisture remained, a tissue was twisted to form a wick, and gently inserted into the canal.

For most procedures, we positioned children supine on the examining table. We collected irrigation effluent with a basin, diaper or disposable blue “chux” pad. For all children age <2 years and some older children, one person (usually the parent) restrained the child's arms, legs, and torso by leaning on the child while a second person immobilized the child's head and a third person performed the procedure. Some older children did not require restraint or head immobilization and thus underwent the procedure in a sitting position.

We established *a priori* stopping rules as follows: upon parent request at any time or if the child (1) was not tolerating the procedure in the opinion of the investigator, (2) experienced injury to the ear canal, (3) was still impacted after 15 min or irrigation with 420 mL. All adverse events (external auditory canal abrasion, TM perforation) were recorded by the research assistant immediately upon completion of the procedure.

Baseline demographic characteristics were collected, as well as wax quality (soft/normal, wet, dry/flaky, hard, or none).

### Outcome Measures

The primary outcome was the reduction in cerumen occlusion, determined by (1) before and after difference and (2) successful cerumen removal after completion of the procedure. The clinician seeing the patient, blinded to the treatment assignment and not part of the study team, characterized the wax and determined the percent cerumen occlusion before and after cleaning. There are no published validated and reliable methods for measuring cerumen content. Young children are generally uncomfortable and uncooperative during ear examinations permitting the examiner only a brief view of the ear canal and making it unreasonable to have a second evaluator verify examinations. For simplicity and expediency, we asked clinicians to estimate and classify cerumen occlusion into five categories: 0, 1–24, 25–49, 50–74, 75–99, and 100%. These categories were assigned numeric values (0–5, respectively) and before and after differences were determined based on these numeric values. Successful cerumen removal was defined as ≤24% occlusion after the procedure.

A research assistant, not involved with the procedure, measured secondary outcomes of time until completion, number of personnel and level of training required to complete the procedure, and parental satisfaction. We recorded the number of personnel and level of training (parents, students, medical assistants, nurses, and physicians) required to position the child, complete the procedure, and perform cleanup. At the study visit and after the procedure, we queried the parents, using a numeric 10-point global rating scale (anchors shown in **Figure 2**), how scary the experience was for their child, how painful the experience was for their child, how their child tolerated the experience, if they would want this procedure done again, how easy the experience was, how they felt about the time it took, and how satisfied they were with their experience.

There are no established outcome measures for children undergoing cerumen removal, which we deemed a distressing but not painful procedure. Data on parent global satisfaction reports and visual analog scales are lacking and self-report is not valid in this age group and established measures for children undergoing painful procedures did not seem appropriate for this study (7).

### Statistics

The sample in this pilot study was limited to a 2-month period of availability of a research assistant. There are no comparative data (1) from which we could estimate a sample size. We analyzed the data using an intention to treat analysis with Stata v14 (StataCorp, College Station, Texas), SAS 9.4 (SAS Institute Inc, Cary, NC) and R v3.5.2. We used Fisher's exact test to compare demographic information, cerumen occlusion, number of stopped procedures per ear, and injuries to the canal. We used ANOVA to compare continuous variables (i.e., time to completion, irrigation volume, number of people required to perform the procedure and parental satisfaction) and Kruskal-Wallis for non-parametric data. We performed univariate logistic mixed models to identify variables (i.e., ear cleaning procedures) associated with successful cerumen removal and to account for potential lack of independence between ears of the same child.

## Results

**Figure 1:**
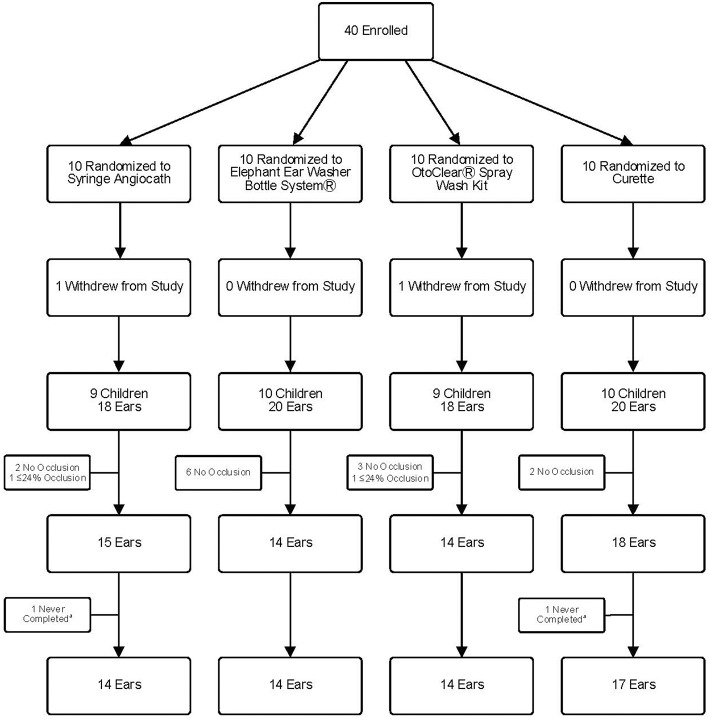
Study Flow Diagram. ^a^Two ears were never completed because the parent requested to stop during cleaning of the previous ear.

We enrolled 40 children; however, two children were withdrawn by parents after randomization but before the procedure started, leaving 38 children (76 ears) (Figure 1). Fifteen ears did not undergo cleaning−13 had no wax and two had <25% occlusion. Parents of two randomized children asked to stop the procedure on the first ear and did not wish to proceed with the second ear despite ≥25% occlusion. Therefore, we present data from ear cleaning procedures on 59 ears (14 SA, 14 EE, 14 OC, and 17 CU). Baseline characteristics of the groups were similar (Table 1).

**Table 1:** Demographic and cerumen characteristics by treatment assignment of 38 children.

**Characteristic**	**SA, *n* = 9**	**EE, *n* = l0**	**OC, *n* = 9**	**CU, *n* = l0**	***P***
**Age (months)**					1.00^a^
6–23	4 (44%)	5 (50%)	5 (56%)	5 (50%)	
24–47	3 (33%)	3 (30%)	2 (22%)	2 (20%)	
48–71	2 (22%)	2 (20%)	2 (22%)	3 (30%)	
Median [IQR]	27.2 [14.4, 41.5]	23.2 [15.6, 41.1]	23.6 [16.7, 35.7]	27.1[11.5, 49.1]	0.99^b^
**Gender**					0.94^a^
Male	5 (56%)	4 (40%)	4 (44%)	4 (40%)	
Female	4 (44%)	6 (60%)	5 (56%)	6 (60%)	
**Race**					0.60^a^
African-American	7 (78%)	8 (80%)	7 (78%)	9 (90%)	
White	1(11%)	2 (20%)	0 (0%)	1(10%)	
Other	1(11%)	0 (0%)	2 (22%)	0 (0%)	
**Health insurance**					0.14^b^
Public	8 (89%)	8 (80%)	5 (56%)	10 (100%)	
Private	1(11%)	2 (20%)	3 (33%)	0 (0%)	
Unknown	0 (0%)	0 (0%)	1(11%)	0 (0%)	
**Cerumen type (L/R)**					0.27^a^/0.23^a^
Soft/normal	7 (78%)/7 (78%)	6 (60%)/6 (60%)	2 (22%)/(33%)	3 (30%)/4 (40%)	
Wet	0 (0%)/0 (0%)	0 (0%)/0 (0%)	3 (33%)/2 (22%)	1(10%)/1(10%)	
Dry/flaky	1(11%)/1(11%)	0 (0%)/0 (0%)	1(11%)/2 (22%)	2 (20%)/2 (20%)	
Hard	0 (0%)/0 (0%)	1(10%)/1(10%)	1(11%)/1(11%)	2 (20%)/3 (30%)	
None	1(11%)/1(11%)	3 (30%)/3 (30%)	2 (22%)/1(11%)	2 (20%)/0 (0%)	

SA, Syringe with attached angiocath tubing; EE, Elephant Ear Washer Bottle System^®^ OC- Otoclear^®^ Spray Wash Kit; CU, metal curette; n, number of children; L, left ear; R, right ear.

a P values determined by Fisher's exact test.

b *P-values determined by Kruskai-Wallis test*.

The difference in cerumen occlusion before and after removal was similar (*P* = 0.32) among the four groups (Table 2). Overall, 36/59 (61%) ears improved to <25% cerumen occlusion (successful cerumen removal) after cleaning and the four procedures were not significantly different (Supplementary Table 1, *P* = 0.67). Wax quality, ear side, and the interaction term between wax quality and ear side did not predict successful cerumen removal (all *P* > 0.40, data not shown). Median procedure time was significantly different (*P* = 0.04) with the SA method taking the most time (4.7 min) and CU taking the least time (1.7 min). Of 59 procedures, 23 (39%) were stopped according to *a priori* rules. Notably, the SA procedure had significantly (*P* < 0.01) more stops than the other methods, especially due to parent request or investigator judgement. The volume used for the three irrigation procedures and number of people needed for positioning were not significantly different. All procedures required three people—a parent to position the child, a second parent, family member, medical assistant, or nurse to immobilize the head and one person to do the procedure. A clinician (doctor or nurse practitioner) did the curetting and a medical assistant or clinic nurse did all irrigation procedures. Cleanup was done by the same clinician, medical assistant, or nurse who did the procedure. Three adverse events were reported. Three children had small abrasions and minimal bleeding in their ear canals from their procedures—two from the SA and one from the OC. After the cerumen removal procedure, two children were diagnosed with AOM.

**Table 2:** Cerumen removal results in 59 ears.

	**SA, *n* = 14**	**EE, *n* = 14**	**OC, *n* = 14**	**CU, *n* = 17**	***P***
**Before/after difference in occlusion category**^**a**^					0.32^c^
−2	0 (0%)	1(7%)	0 (0%)	0 (0%)	
0	4 (29%)	1(7%)	2 (14%)	4 (24%)	
1	3 (21%)	1(7%)	2 (14%)	3 (18%)	
2	1(7%)	5 (36%)	5 (36%)	7 (41%)	
3	1(7%)	3 (21%)	0 (0%)	2 (12%)	
4	4 (29%)	2 (14%)	2 (14%)	1(6%)	
5	1(7%)	1(7%)	3 (21%)	0 (0%)	
Successful cerumen removal (%)^b^	6 (43%)	9 (64%)	10 (71%)	11(65%)	0.67^e^
Time from initiation to completion (Median [IQR], mins)	4.7 [2.2,8.2]	1.9 [1.1, 3.7]	2.5 [1.7,3.9]	1.7 [1.3, 2.2]	0.04^d^
**Reasons for stopped procedures**					
Time limit	0	0	0	0	
Volume	2	3	2	0	
Parent request	6	1	2	2	
Investigator stopped	3	0	0	2	
Total	11(79%)	4 (29%)	4 (29%)	4 (24%)	<0.01^c^
Number of injured canal(s) (*N* = 38 children)	2 (22%)	0 (00/o)	1(11%)	0 (0%)	0.13^c^
Mean (sd) volume used (ml)	242.9 (131.5)	255.4 (128.5)	236.8 (143.7)	N/A	0.93
**Mean (sd) number of people needed (*****N*** **=** **38 children)**					
Positioning	1.9 (0.3)	1.9 (0.6)	1.9 (0.3)	1.8 (0.4)	0.92
Procedure	1(0.0)	1(0.0)	1(0.0)	1(0.0)	

SA, Syringe with attached angiocath tubing; EE, Elephant Ear Washer Bottle System^®^ OC, Otoclear^®^ Spray Wash Kit; CU, metal curette; n, number of ears cleaned.

a Percent occlusion ranked as 0–5 ordinal scale: 0 (0), 1–24 (1), 25–49 (2), 50–74 (3),75–99 (4), 100 (5).

b Ears with <25% occlusion after cleaning.

c P-values determined by the Fisher's exact test.

d P-values determined by the Kruskai-Wallis test.

e *Overall P values determined by logistic mixed model*.

**Figure 2:**
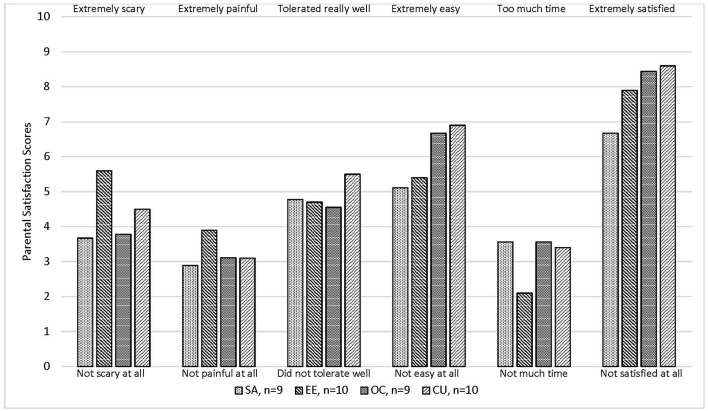
Mean parental satisfaction score for 38 children who underwent cerumen removable procedure*. SA, Syringe with attached angiocath tubing; EE, Elephant Ear Washer Bottle System^®^; OC, Otoclear^®^ Spray Wash Kit. *P-values were not statistically significant (*P* < 0.05) by the Kruskal-Wallis test.

Figure 2 shows that parental satisfaction scores were not significantly different by treatment. Overall, parents felt the procedures were relatively painless, easy and quick (33, 28 and 33 of 38 gave favorable ratings of ≤5, ≥5, and ≤5, respectively, on a scale of 0–10).

## Discussion

In this randomized clinical trial, we found that irrigation methods were comparable to curetting cerumen-occluded ears in young children. Reduction in cerumen occlusion, successful removal and parental satisfaction scores were not significantly different. Curetting took less time to perform; however, unlike irrigation methods it required the presence of a trained clinician.

Our findings should be interpreted in light of several limitations; chiefly, the small sample size and four treatment groups in this pilot study may have prevented detecting some important differences (type II error). Our sample size was determined by clinician referrals and the availability of a research assistant for 2 months. Having two outcome measures for cerumen removal (before and after difference and successful cerumen removal) prevented the calculation of a combined *post-hoc* power calculation. However, a *post-hoc* analysis for the successful cerumen removal outcome demonstrated the sample size needed would be 59 ears randomized to each procedure in order to reach a power of 80% (alpha = 0.05, two-sided) (8). Furthermore, the clinicians who estimated cerumen occlusion before and after the procedures did not undergo standardized training or inter-rater reliability testing. Finally, the parent satisfaction instrument has not undergone psychometric testing. Despite these limitations, we built on the very limited previous literature. To our knowledge, this is the first study in any age group comparing different methods of cerumen removal (1). Strengths of this study, not addressed in previous published reports, include use of examiners who were blinded to treatment assignment, assessing the change in cerumen occlusion for each ear before and after the procedure, documenting duration of procedures and measuring parental impressions—important because these children were too young to self-report procedural discomfort.

Success rates for cerumen removal (defined by ≤24% occlusion in our study) were 65% for the CU and 71% for the OC and EE which are similar to previous studies of irrigation that included children (9, 10). Though the SA only succeeded in 43% of children in our study, before and after differences were similar for all methods. A large proportion (39%) of procedures were stopped at some point, most commonly due to parental request, which likely reduced success rates for all procedures. Most children in our study were not ill and therefore the parental tolerance for any discomfort to their child may have been lower than if their child had a clear clinical need to visualize the TM.

Among irrigation methods, the SA resulted in significantly more stopped procedures, took more time due to the need to refill the syringe multiple times (despite the large number of stopped procedures), and resulted in two small abrasion injuries to the external canal. Subjectively, the SA exerted a more forceful stream of water than the EE or OC which, if unintentionally aimed directly at the TM, may have caused some children discomfort. It was also difficult to control the depth and placement of the flexible angiocath tip while simultaneously exerting enough force to depress the plunger which sometimes required two hands. Based on these findings, we would not recommend the SA for young children. Although there was one abrasion to the external auditory canal of a child in the OC group, the speculum-like soft plastic tip seems very safe—it cannot enter too far, and it aims the streams of water obliquely to the sides of the canal. This child may have been injured by the edge of an ear speculum when a medical assistant checked to assess the need to continue. No other adverse events were noted.

There are only two randomized trials (9, 10) of cerumen removal that included children, enrolling 13 and 92 children age <5 years, respectively. These studies compared different cerumenolytics rather than methods of cerumen removal. Children in these studies received a cerumenolytic in the ear canal for 15 min followed by irrigation using the SA method. Both studies demonstrated that the irrigation, rather than the cerumenolytic softening agents was the most effective intervention. A systematic review of clinical trials and a test tube study of cerumen pellet dissolution concluded that cerumenolytics were no better than water or saline (5, 11).

In one case series of the OC in an older group of children age <11.5 years, investigators achieved ≥80% visibility of the TM in all children but needed >1 washing containing 205 mL of water in 39% of children. Another case series of older children (mean age 7.5 years) demonstrated 91.9% rate of complete removal of cerumen using a continuous flow, pressure-regulated water irrigation device (12). These findings suggest a higher success rate in children may be possible if they can cooperate long enough to receive sufficient irrigation volume. Mean irrigation volume in our study was approximately 250 mL.

We found that irrigation procedures were easier to perform in children age <2 years when they were placed supine on the examining table rather than in their parents' lap. Having the child sit in the parent's lap facing away from the parent with the parent restraining the child's head, torso, and extremities was ineffective because the child could move too much. Having the child lie supine on the examining table with a parent restraining the torso and extremities and another person immobilizing the head gave better control. However, this position posed some equipment issues. Commercially available water collection basins used with the OC and EE work well for cooperative seated children, but rise above the auditory canal for most children aged <2 years who are supine. Instead, we used a diaper and/or a blue “chux” pad placed under the child's head which could soak up 500 mL and worked relatively well. The second issue was that the OC and EE each have a reservoir connected to the hand-held squeeze-trigger that drew water from the bottom of the reservoir via a tube. When the child was supine in the middle of the exam table, the squeeze bottle had to be turned horizontal, and the tube at the bottom of the reservoir would run dry before the reservoir was completely empty. To address this, we had to move children to the edge of the exam table so the squeeze bottle could be vertical. Some simple product modifications would optimize these devices for use in young children.

## Conclusions

This pilot study suggests that irrigation and curettage are comparable methods for cerumen removal in children age <6 years. The SA had some drawbacks compared to other methods. Irrigation techniques were feasible, with some positioning and equipment modifications required for the youngest children, and relatively safe and well-tolerated. With proper training a non-clinician can perform irrigation to improve TM visualization in young children—a significant advantage over curetting which requires a clinician. Future studies comparing cerumen removal methods should include a larger sample of the youngest children age <2 years and exclude the SA method. Product modifications should occur to make irrigation methods easier to use for the youngest children, who have the highest incidence of AOM. Finally, more rigorous, methods of cerumen measurement should be developed.

## Ethics Statement

This study was carried out in accordance with the recommendations of the NIH Good Clinical Practice with written informed consent from the parent of all subjects (the subjects were children and too young to give assent or consent). All parents of subjects gave written informed consent in accordance with the Declaration of Helsinki. The protocol was approved by the Institutional Review Board of the University of Pittsburgh.

## Author Contributions

TS participated in study concept and design, designing the database and data collection form, acquisition and analysis of data, and drafting/revising the manuscript. HL contributed to analysis of data and drafting/revising the manuscript. CC participated in acquisition and analysis of data and drafting/revising the manuscript. NS participated in study concept and design, designing the database and the data collection form, acquisition and analysis of data, and drafting/revising the manuscript. All authors approved the final manuscript as submitted and agree to be accountable for all aspects of the work.

### Conflict of Interest Statement

The authors declare that the research was conducted in the absence of any commercial or financial relationships that could be construed as a potential conflict of interest.

## Abbreviations

TM: Tympanic membrane
AOM: Acute otitis media
SA: Syringe with attached angiocath tubing
EE: Elephant Ear Washer Bottle System^®^
OC: Otoclear^®^ Spray Wash Kit
CU: metal curette.

## References

[1] Schwartz SR Magit AE Rosenfeld RM Ballachanda BB Hackell JM Krouse HJ. Clinical practice guideline (update): earwax (cerumen impaction) executive summary. Otolaryngol Head Neck Surg. (2017) 156:14–29. 10.1177/0194599816678832 28045632

[2] Lieberthal AS. Acute otitis media guidelines: review and update. Curr Allergy Asthma. (2006) 6:334–41. 10.1007/s11882-006-0069-5 16822388

[3] McCarter DF Courtney AU Pollart SM. Cerumen impaction. Am Fam Physician. (2007) 75:1523–28. 17555144

[4] Shaikh N Hoberman A Kaleida PH Ploof DL Paradise JL. Videos in clinical medicine. Diagnosing otitis media–otoscopy and cerumen removal. N Engl J Med. (2010) 362:e62. 10.1056/NEJMvcm0904397 20484393

[5] Burton MJ Doree C Ear drops for the removal of ear wax. Cochrane Database Syst Rev. 2009:CD004326. 10.1002/14651858.CD004326.pub2 19160236

[6] Abdala C Keefe DH Morphological and functional ear development. In: Werner L Fay R Popper A editors, Human Auditory Development. Springer Handbook of Auditory Research. Vol. 42 New York, NY: Springer (2012). 10.1007/978-1-4614-1421-6_2

[7] McGrath PJ Walco GA Turk DC Dworkin RH Brown MT Davidson K. Core outcome domains and measures for pediatric acute and chronic/recurrent pain clinical trials: PedIMMPACT recommendations. J Pain. (2008) 9:771-783. 10.1016/j.jpain.2008.04.007 18562251

[8] Diggle P Liang K Zeger SL Analysis of Longitudinal Data. Oxford: Clarendon Press (1999).

[9] Singer AJ Sauris E Viccellio AW. Ceruminolytic effects of docusate sodium: a randomized, controlled trial. Ann Emerg Med. (2000) 36:228–32. 10.1067/mem.2000.109166 10969225

[10] Whatley VN Dodds CL Paul RI. Randomized clinical trial of docusate, triethanolamine polypeptide, and irrigation in cerumen removal in children. Arch Pediatr Adolesc Med. (2003) 157:1177–80. 10.1001/archpedi.157.12.1177 14662569

[11] Saxby C Williams R Hickey S. Finding the most effective cerumenolytic. J Laryngol Otol. (2013) 127:1067–70. 10.1017/S0022215113002375 24148313

[12] Propst EJ George T Janjua A James A Campisi P Forte V. Removal of impacted cerumen in children using an aural irrigation system. Int J Pediatr Otorhinolaryngol. (2012) 76:1840–3. 10.1016/j.ijporl.2012.09.014 23040963

