# Supplementary material for: Randomized Trial of Irrigation and Curetting for Cerumen Removal in Young Children
Source: Front Pediatr. 2019 Jun 6;7:216. doi: 10.3389/fped.2019.00216 (PMC6563688; doi:10.3389/fped.2019.00216)
Supplement: Supplementary file 1 [file Table_1.pdf]

Supplemental Table 1. Odds Ratio for Successful Cerumen Removal from Logistic Mixed Model in 59 Ears.

	β	OR	95% CI[b]		p[b]
Ear cleaning procedures[a]					
SA vs. EE	-0.86	0.42	0.03	6.78	>0.99
SA vs. OC	-1.15	0.32	0.02	5.51	>0.99
SA vs. CU	-0.82	0.44	0.03	6.38	>0.99
EE vs. OC	-0.29	0.75	0.04	13.34	>0.99
EE vs. CU	0.04	1.04	0.07	15.47	>0.99
OC vs.CU	0.33	1.39	0.09	22.70	>0.99

a. Overall *P* value 0.67

b. With Bonferroni adjustment

We ran a univariate logistic mixed model to test if there was an association between ear cleaning procedures and successful cerumen removal. We applied Bonferroni adjustment to the results, which showed no difference among four ear cleaning procedures (all p>0.99).
